# Supplementary material for: Spatially conserved motifs in complement control protein domains determine functionality in regulators of complement activation-family proteins
Source: Commun Biol. 2019 Aug 5;2:290. doi: 10.1038/s42003-019-0529-9 (PMC6683126; doi:10.1038/s42003-019-0529-9)
Supplement: Supplementary file 1 — Supplementary information [file 42003_2019_529_MOESM1_ESM.pdf]

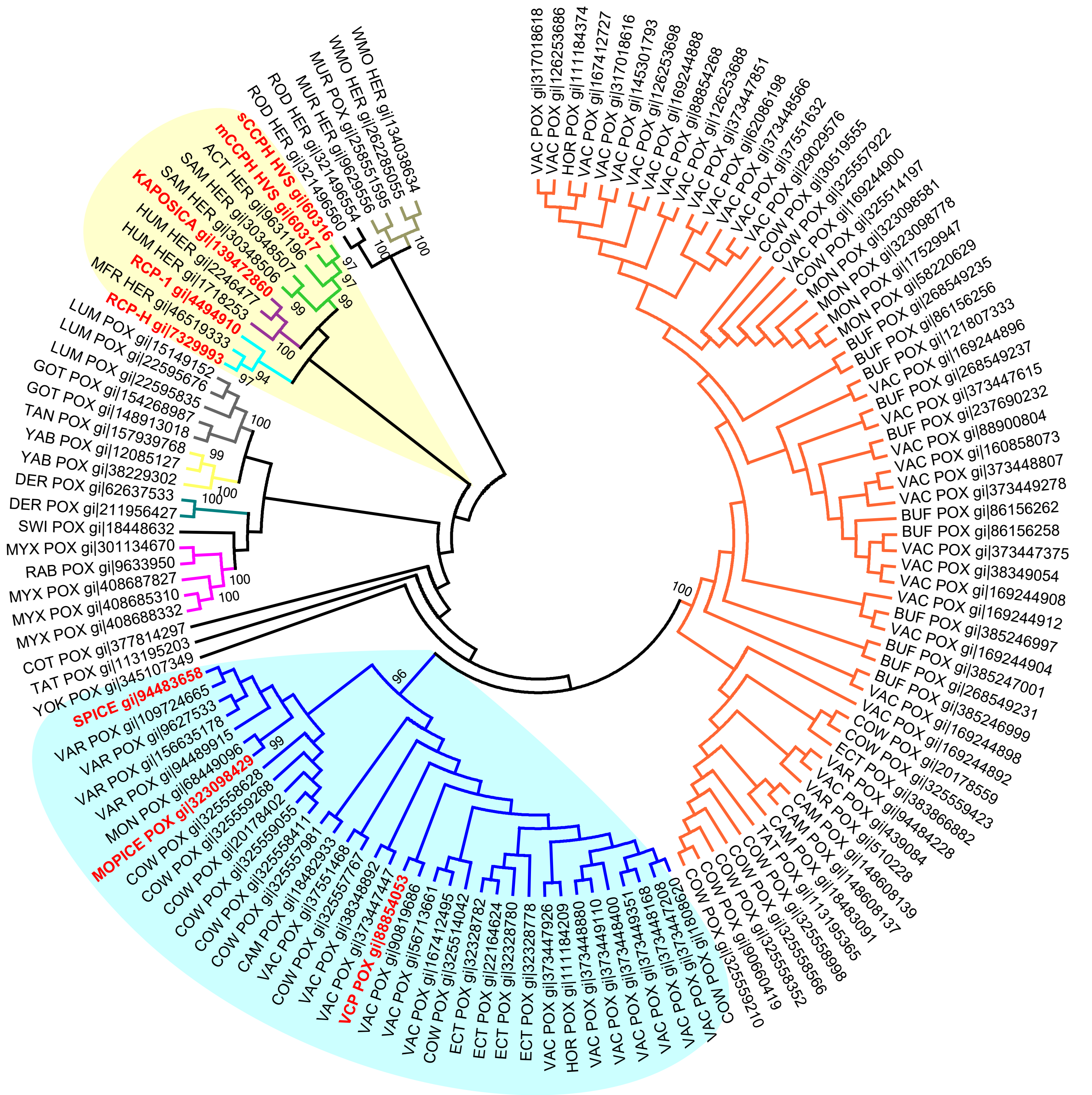

Supplementary Figure 1: Selection of viral sequences for MEME analysis. The selection of sequences for motif analysis by MEME was based on phylogenetic analysis of CCP domain containing sequences. The phylogenetic tree was constructed using the Neighbor-Joining method with 1000 bootstrap replicates. The figure shows the example of a phylogenetic tree of viral sequences where clades (>90 bootstrap value) are highlighted with different colors. The clades (shaded) with functionally characterized sequences (highlighted in red font) were selected for further analysis by MEME.

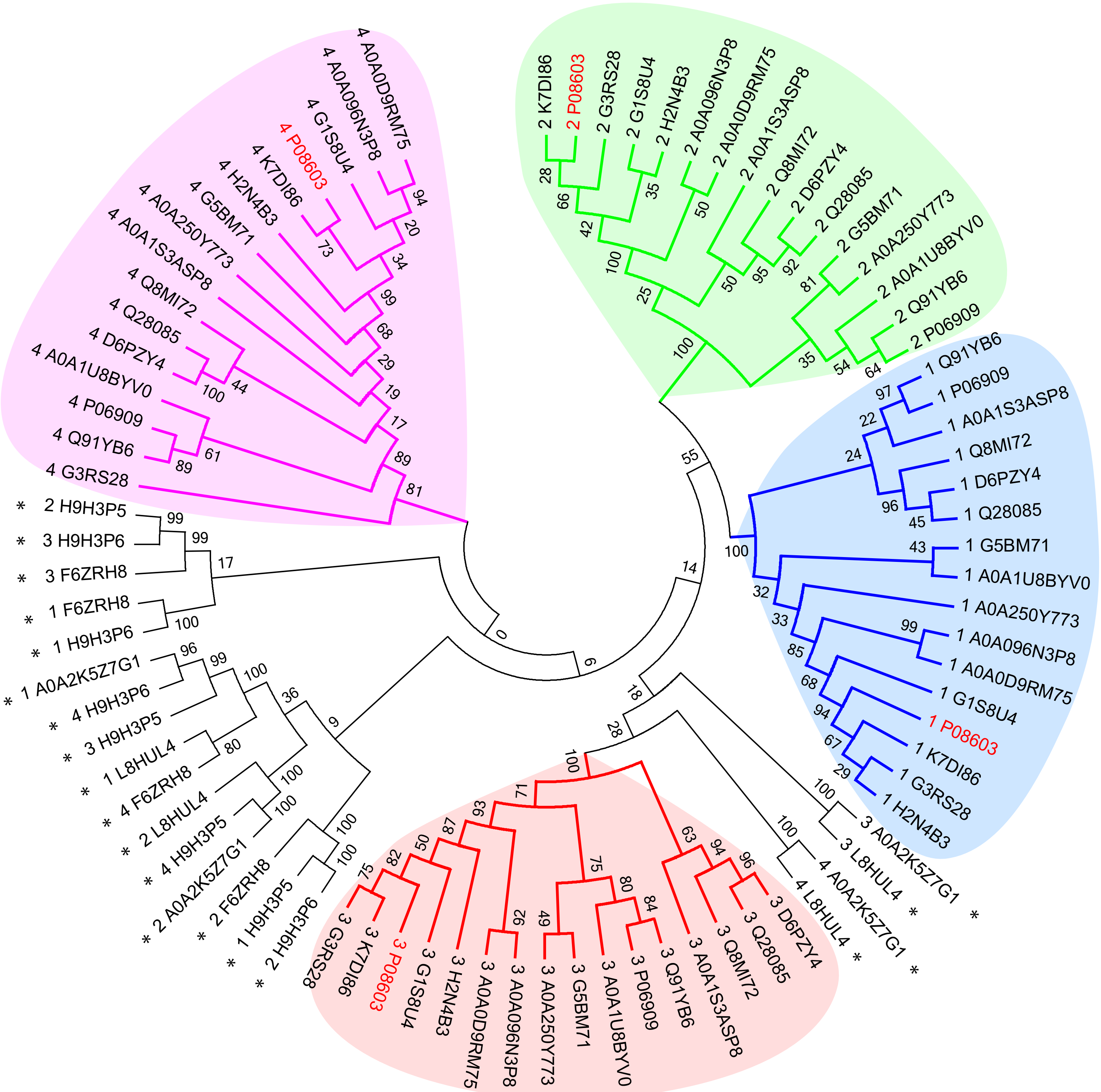

Supplementary Figure 2: Selection of mammalian sequences for MEME analysis. The selection of mammalian sequences for motif analysis by MEME was based on phylogenetic analysis of individual CCP domains. The phylogenetic tree was constructed using the Neighbor-Joining method with 1000 bootstrap replicates. The figure shows the example of a phylogenetic tree of individual CCPs of CR1 sequences where clades (>90 bootstrap value) are highlighted with different colors. Here, putative regulatory CCPs that formed a clade (shaded) with the respective functionally characterized human CCPs (highlighted in red font) were selected and rest were removed (marked with an asterisk).

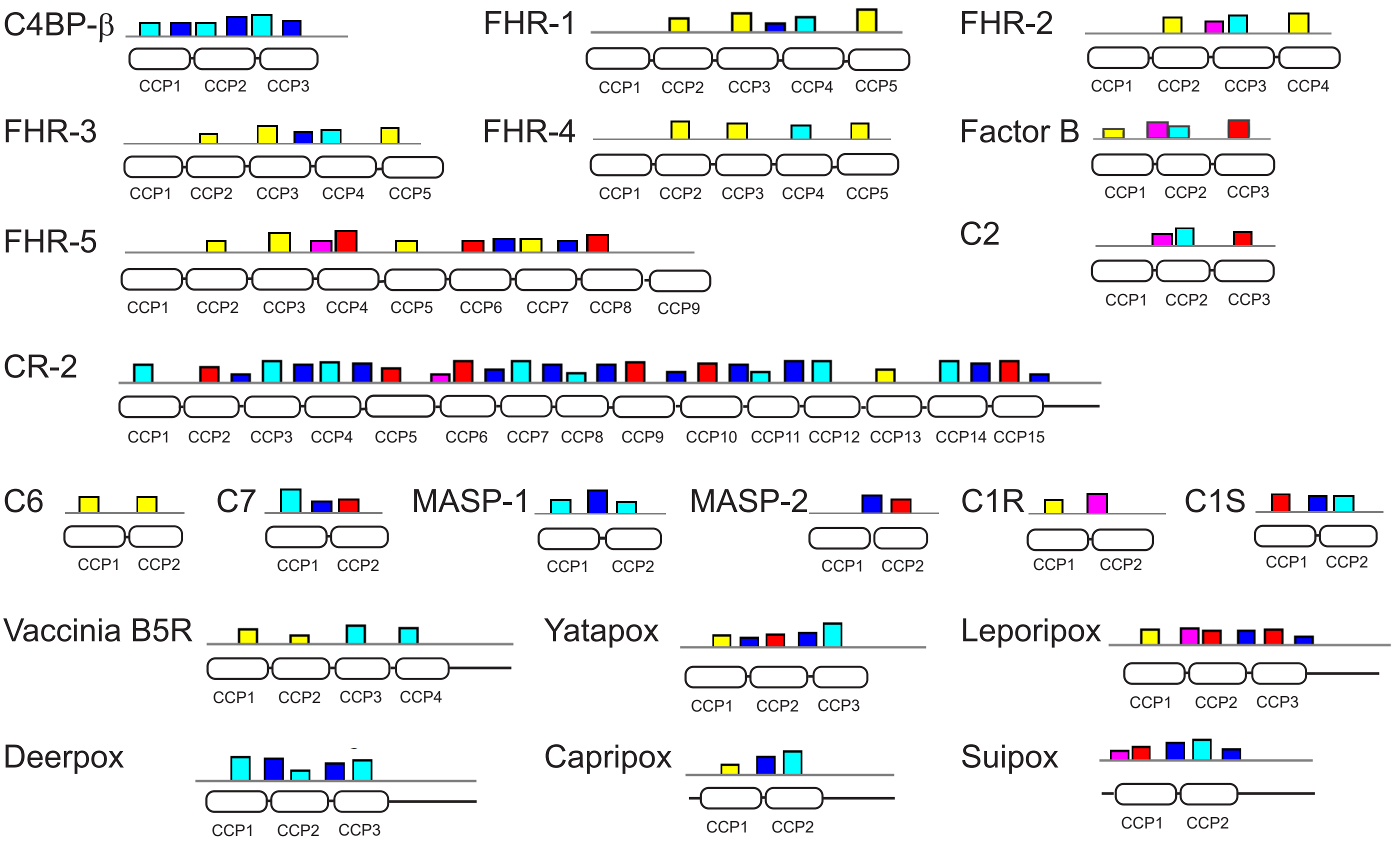

Supplementary Figure 3: Non-regulatory human and viral RCA proteins lack the conserved motif pattern. MAST scanning of non-regulatory RCA protein sequences by 5 motifs showed a random motif distribution. For each protein, the top lane represents motifs identified by MAST scanning and the bottom lane represents the respective CCP domain. An absence of shading in the CCP domains indicates the lack of the signature motif pattern. C4BP-β, C4b-binding protein- β chain; FHR-1, Complement factor H-related protein-1; FHR-2, Complement factor H-related protein-2; FHR-3, Complement factor H-related protein-3; FHR-4, Complement factor H-related protein-4; FHR-5, Complement factor H-related protein-5; C2, complement component 2; C6, complement component 6; C7, complement component 7; CR-2, Complement receptor-2; MASP-1, MBL-associated serine protease-1; MASP-2, MBL-associated serine protease-2.

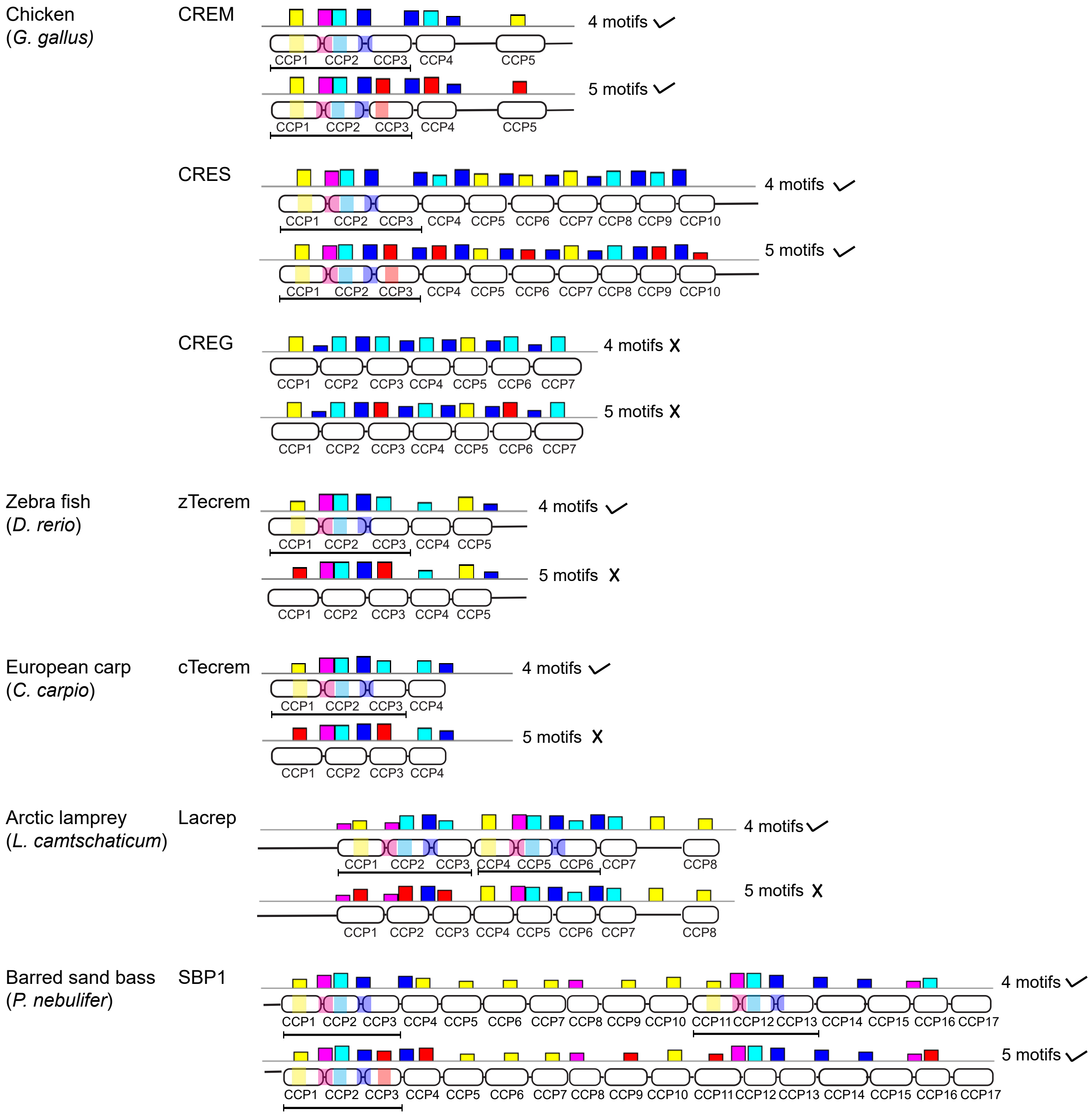

Supplementary Figure 4: The signature 4-motif pattern (M5-M3-M1-M2) based annotation exhibits higher sensitivity for non-mammalian proteins. Functionally characterized regulatory proteins of Chicken (Gallus gallus), Zebra fish (Danio rerio), European carp (Cyprinus carpio), Arctic lamprey (Lethenteron camtschaticum) and Barred sand bass (Paralabrax nebulifer) scanned by 4 and 5 motifs. For each protein, the top and bottom lanes represent MAST scanning results with 4 and 5 motifs, respectively. The position of signature motif patterns M5-M3-M1-M2 and M5-M3-M1-M2-M4 are shown by shading on CCP domains with respective motif colours and the CCP domains implicated in function are marked by a horizontal line. The presence of a signature motif pattern in protein is indicated by a tick. CREM, Complement regulatory membrane protein of chicken; CRES, Complement regulatory secretory protein of chicken; CREG, Complement regulatory GPI-anchored protein of chicken; Tecrem, teleost complement-regulatory membrane protein; zTecrem, zebrafish Tecrem; cTecrem, carp Tecrem; Lacrep, lamprey complement regulatory protein; SBP1, sand bass protein 1.

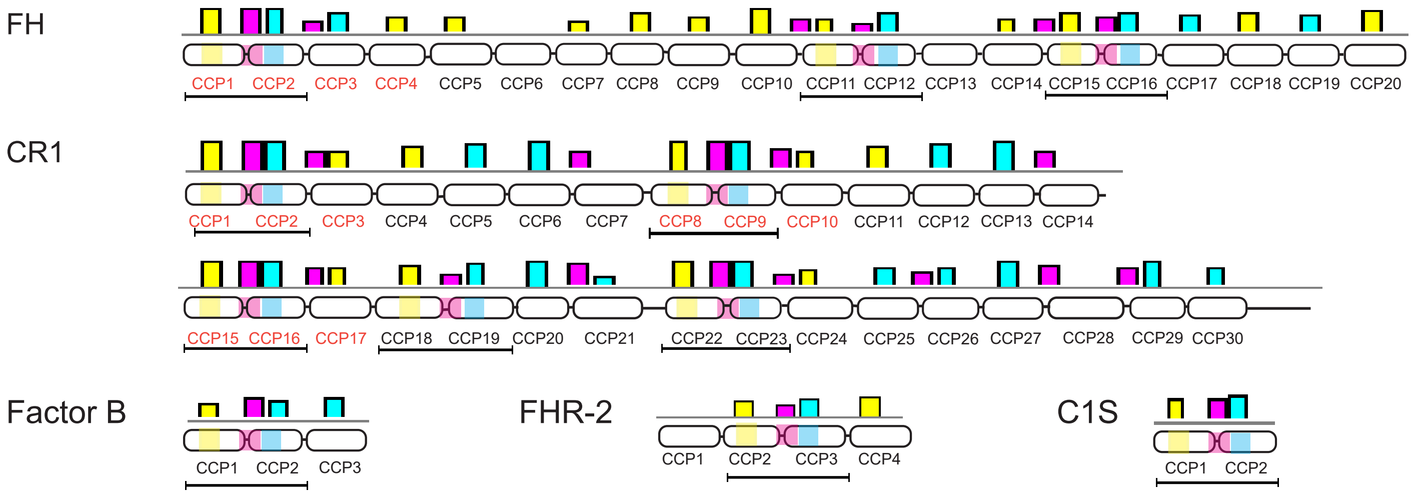

Supplementary Figure 5: MAST scanning of RCA proteins with 3-motif pattern (M5-M2-M1). MAST scanning of with 3-motif pattern (M5-M3-M1) resulted in imprecise recognition of non-regulatory CCP domains as complement regulatory sites. A horizontal line indicates the presence of a 3-motif pattern in the implicated CCP domains. FH, Complement factor H; CR1, Complement receptor 1; FHR-2, Complement factor H-related protein-2.

Supplementary Fig. 6a

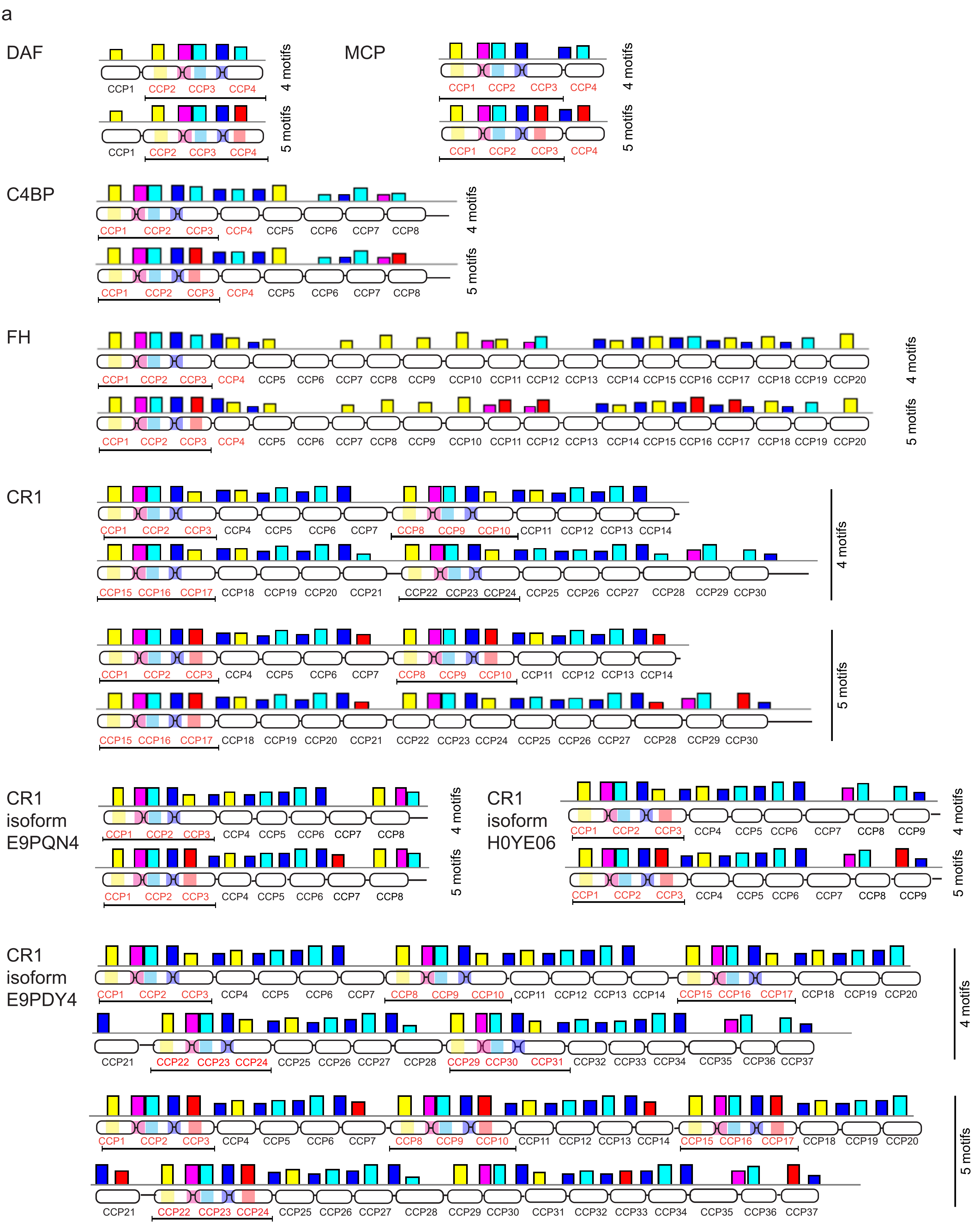

Supplementary Fig. 6b

b

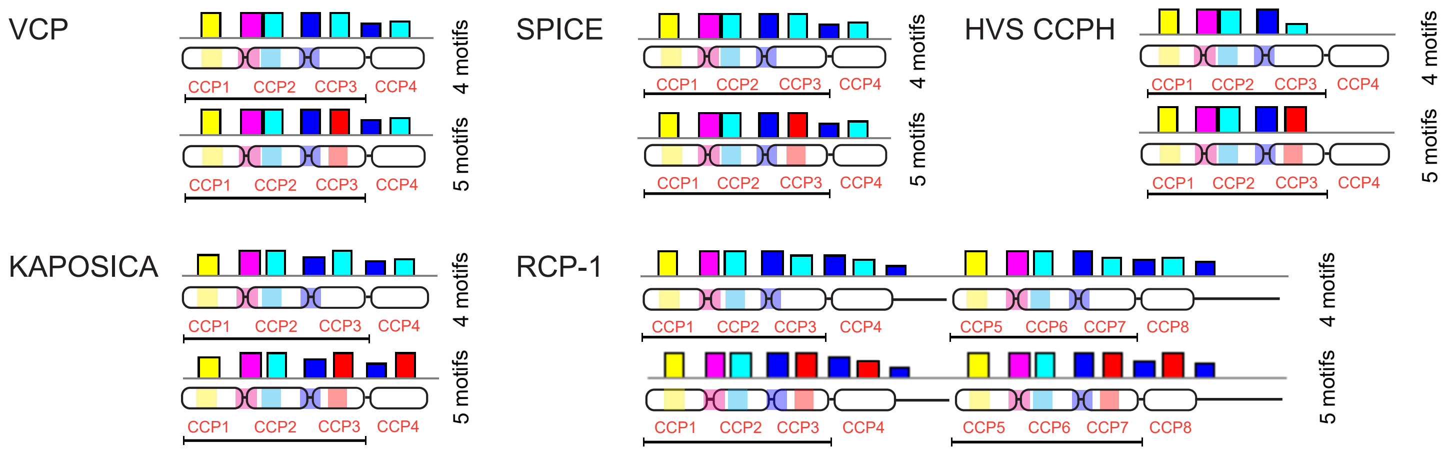

Supplementary Figure 6: MAST scanning of RCA proteins with 4- and 5-motif pattern. MAST scanning performed by 4 and 5 motifs in human **(a)** and viral **(b)** regulators. For each regulator, top and bottom lanes represent MAST scanning based on 4 motifs and 5 motifs, respectively. The position of signature motif patterns M5-M3-M1-M2 and M5-M3-M1-M2-M4 are shown by shading on CCP domains with respective motif colours and CCP domains implicated in function are marked by a horizontal line.

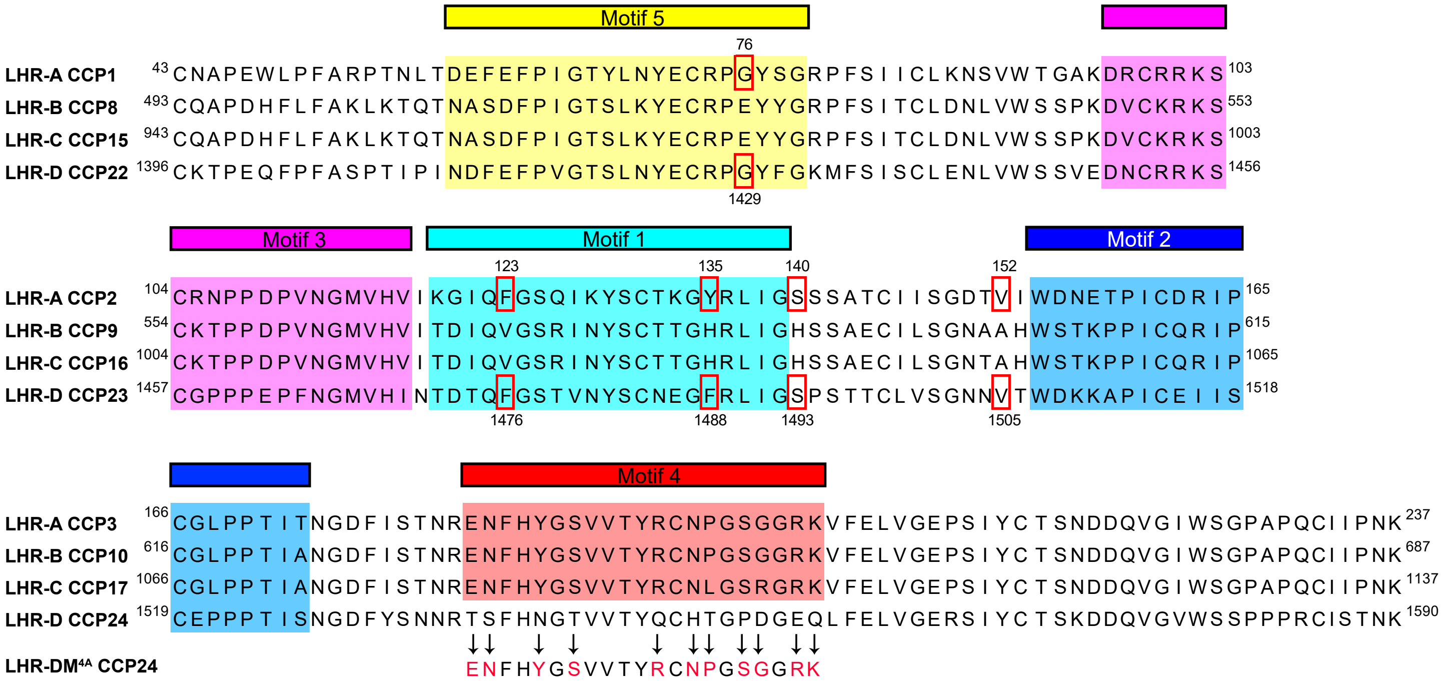

Supplementary Figure 7: LHR-D (CCP22-24) exhibit higher similarity towards LHR-A (CCP1-3) than LHR-B (CCP8-10) and LHR-C (CCP15-17). Motif regions are marked in different colours; Motif 5 (yellow), Motif 3 (pink), Motif 1 (sky blue), Motif 2 (blue) and Motif 4 (red). LHR-A (CCP1-3) residues crucial for DAA are conserved in LHR-D (CCP22-24) (red boxes) as opposed to LHR-B (CCP8-10) and LHR-C (CCP15-17).

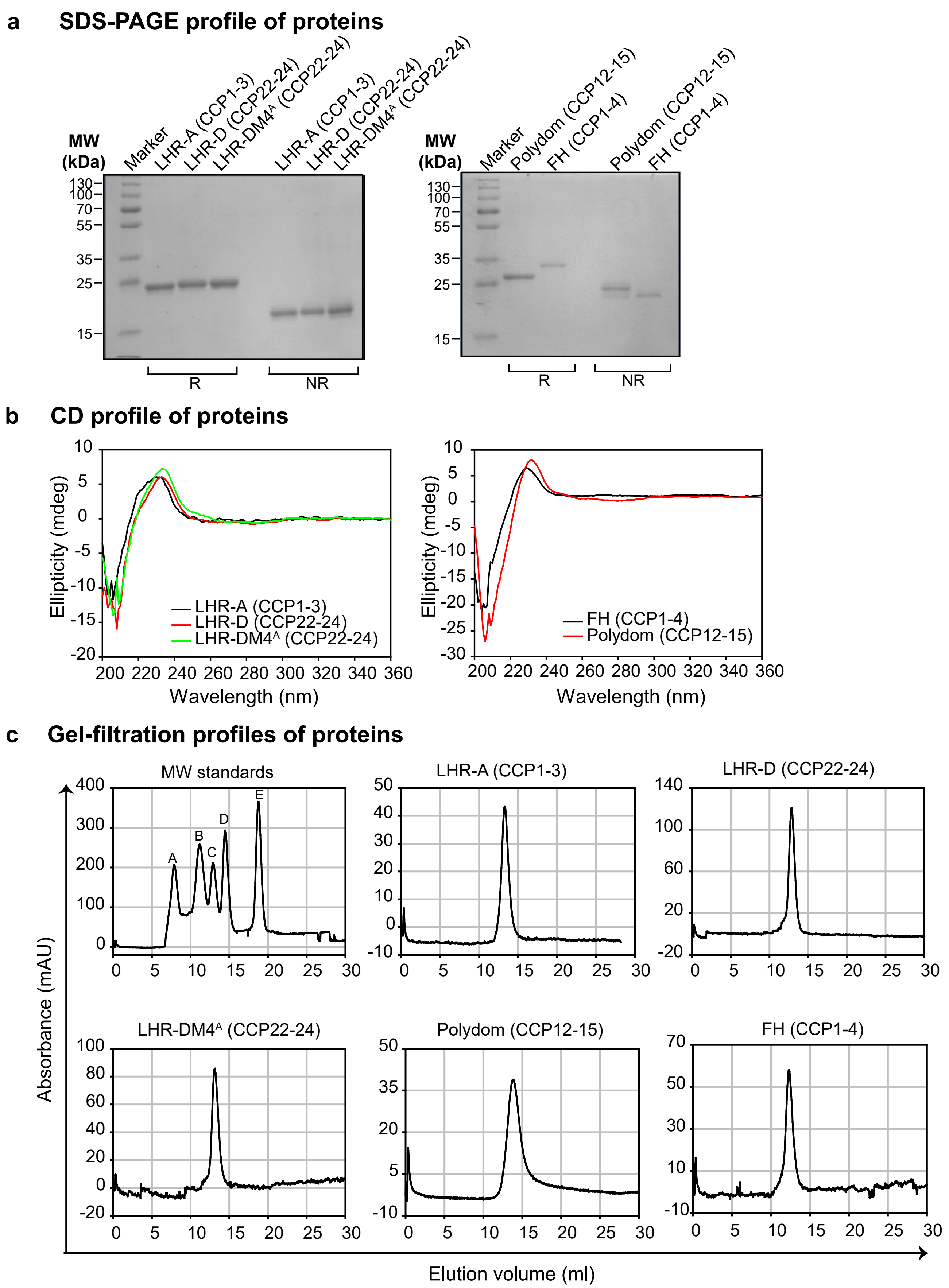

Supplementary Figure 8: Analysis of purified proteins. (a) SDS-PAGE analysis of LHR-A (CCP1-3), LHR-D (CCP22-24), LHR-DM4^A (CCP22-24), polydom (CCP12-14) and FH (CCP1-4). The proteins were run on 12% SDS-PAGE under reducing (R) and non-reducing (NR) conditions. **(b)** CD spectra of LHR-A (CCP1-3), LHR-D (CCP22-24), LHR-DM4^A (CCP22-24) on the left panel and FH (CCP1-4), polydom (CCP12-14) on the right panel. **(c)** Gel filtration analysis of LHR-A (CCP1-3), LHR-D (CCP22-24), LHR-DM4^A (CCP22-24), polydom (CCP12-15) and FH (CCP1-4). MW standards represent known molecular weights protein peaks as below: A, Thyroglobulin (670 kDa); B, Gamma globulin (158 kDa); C, Ovalbumin (44kDa); D, Myoglobin (17 kDa) and E, Vitamin B-12 (1.35 kDa). FH (1-4) was included as a control.

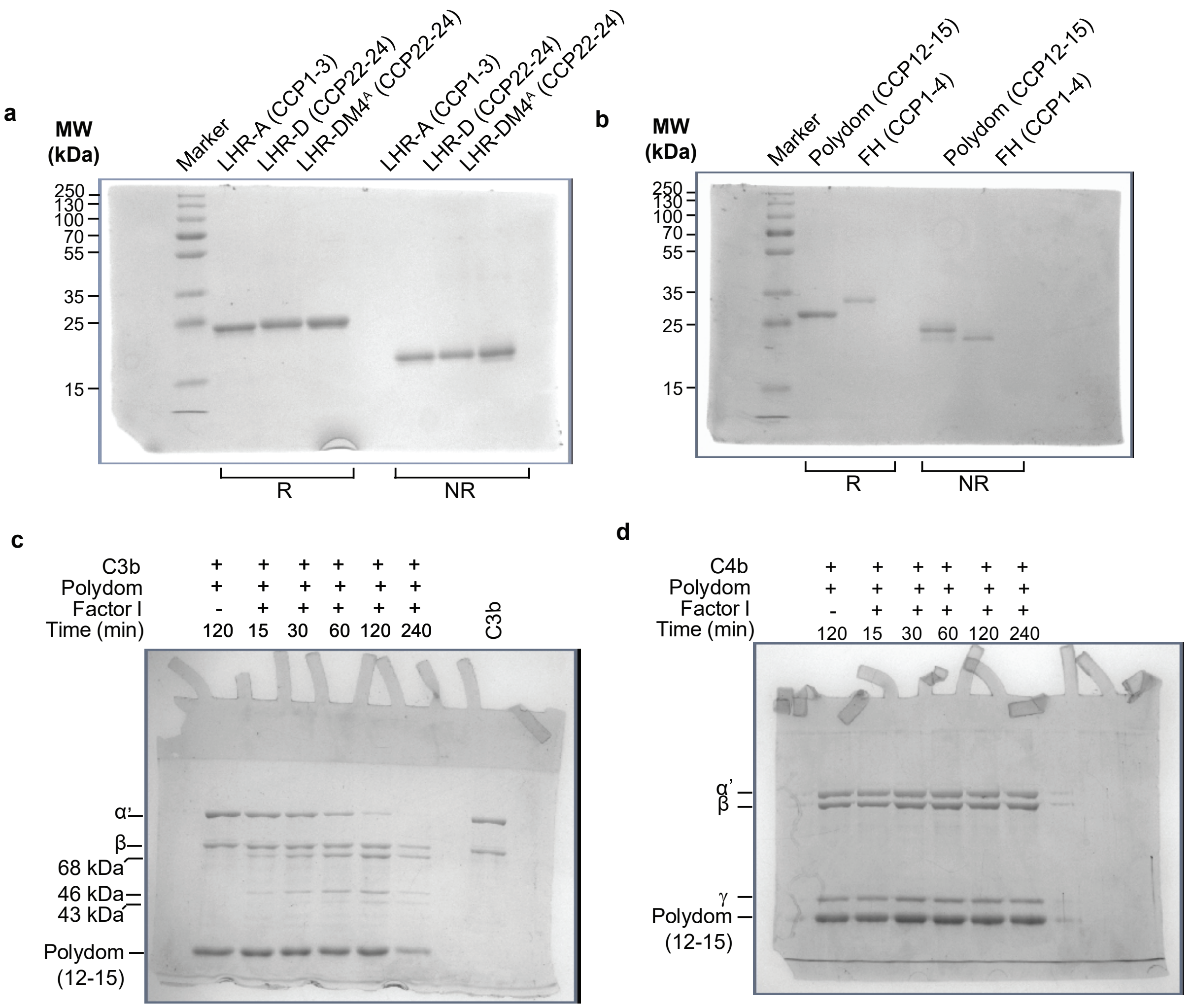

Supplementary Figure 9: Uncropped SDS-PAGE gels of purified proteins and polydom cofactor analysis. a. & b. Uncropped SDS-PAGE gels of LHR-A (CCP1-3), LHR-D (CCP22-24), LHR-DM4^{A} (CCP22-24), polydom (CCP12-14) and FH (CCP1-4) as shwon in **Fig. 4 and 5**. The proteins were run on 12% SDS-PAGE under reducing (R) and non-reducing (NR) conditions. **c. & d.** SDS-PAGE visualization of α'-chain of C3b **(c)** and C4b **(d)** of polydom as shown in **Fig.5.** The 240 min timepoint was not considered in the main figure due to loading problem in **9c**.

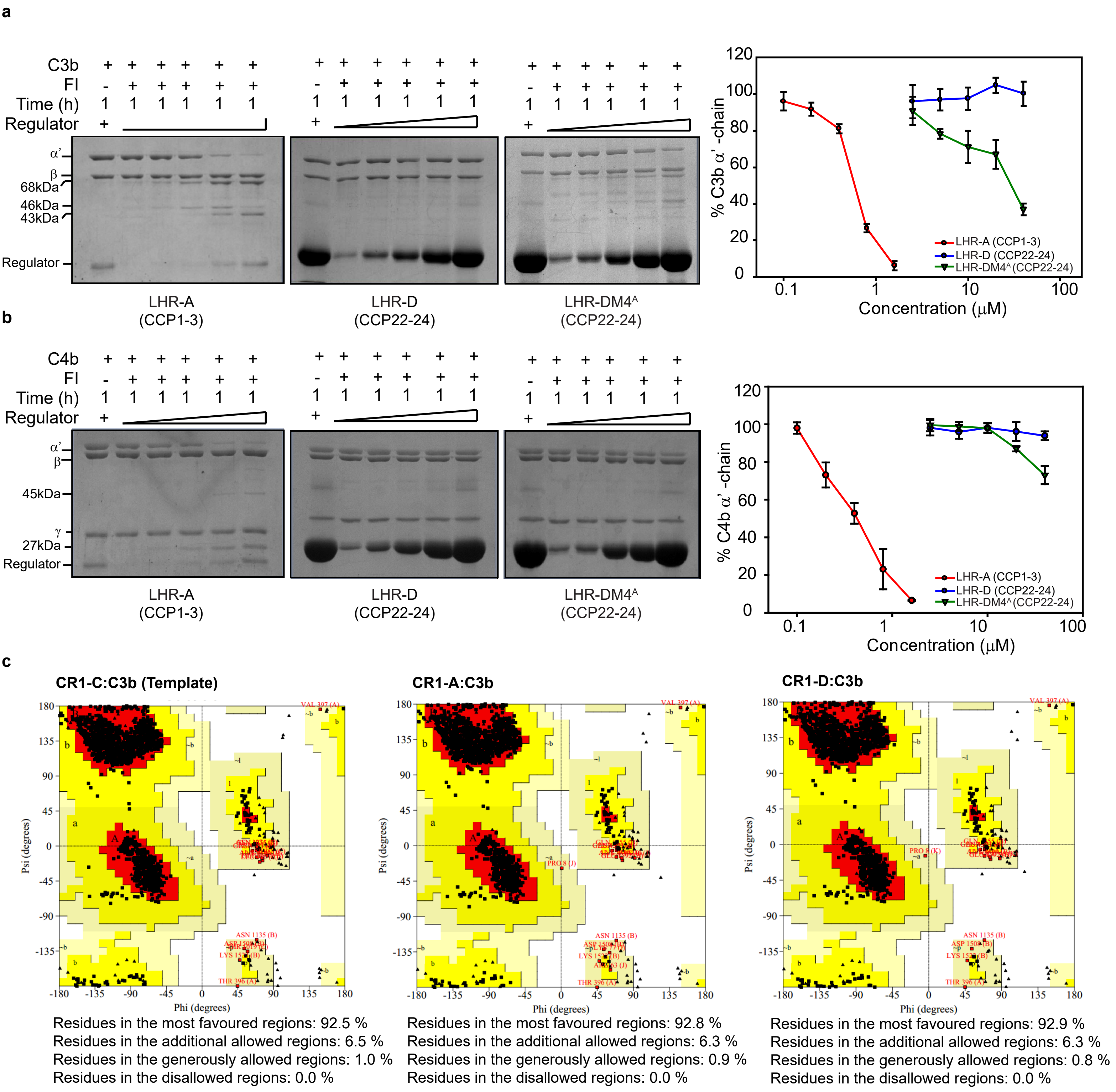

Supplementary Figure 10: Characterization of LHR-DM4^{A} (CCP22-24) and quality analysis of CR1 LHR-A (CCP1-3)-C3b and LHR-D (CCP22-24)-C3b models. (a & b) LHR-DM4^{A} (CCP22-24) gained minimal cofactor activity. Coomassie-stained SDS-PAGE gels for C3b **(a)** and C4b **(b)** cofactor activity of LHR-A (CCP1-3), LHR-D (CCP22-24) and LHR-DM4^{A} (CCP22-24) are shown on the left and represented graphically at right. The cofactor activity of LHR-A (CCP1-3), LHR-D (CCP22-24) and LHR-DM4^{A} (CCP22-24) were assessed by incubating increasing concentrations of each of these regulators with C3b/C4b and FI for an hour at 37°C. The efficiency of the regulator was determined by measuring the cleavage of α'-chain of C4b/C4b. **(c)** Quality analysis of the generated models. Ramachandran plots of homology models of LHR-A (CCP1-3):C3b and LHR-D (CCP22-24):C3b as compared to the template structure of LHR-C (CCP15-17):C3b by using PROCHECK.

a

β2-glycoprotein I (β2-GPI)

b

Polydom (SVEP1)

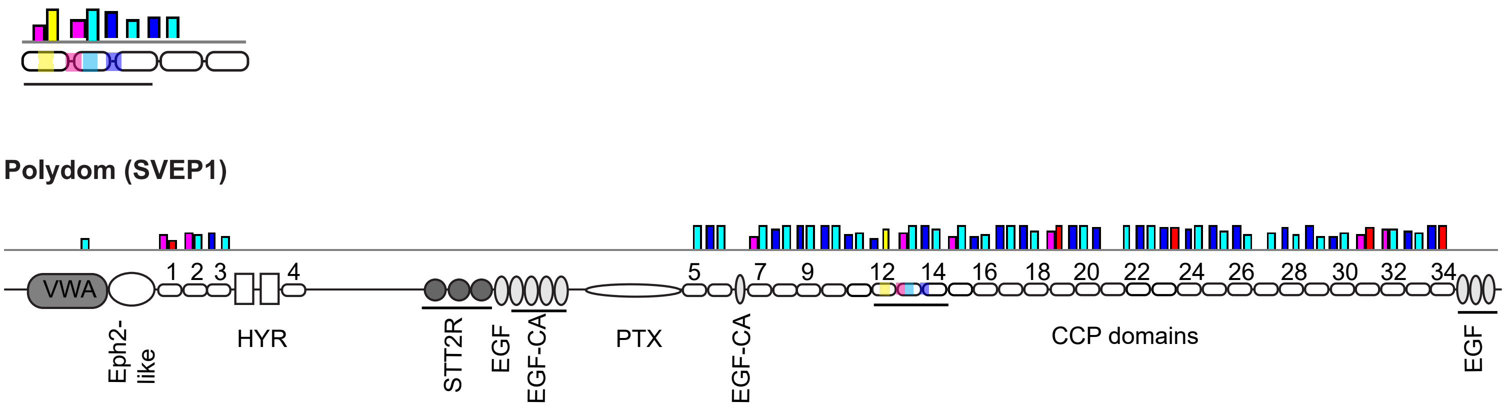

Supplementary Figure 11: Additional CCP-containing human proteins having signature motif pattern. (a) β2-GPI and **(b)** Polydom. Mast scanning result are shown above the domains, and the signature 4 motif pattern in CCP domains (12-14 of Polydom and 1-3 of β2-GPI) is shown by shading on CCP domains with respective motif colours. The CCP domains implicated in function in β2-GPI and those that are likely to have function in polydom are marked by a horzontal line.

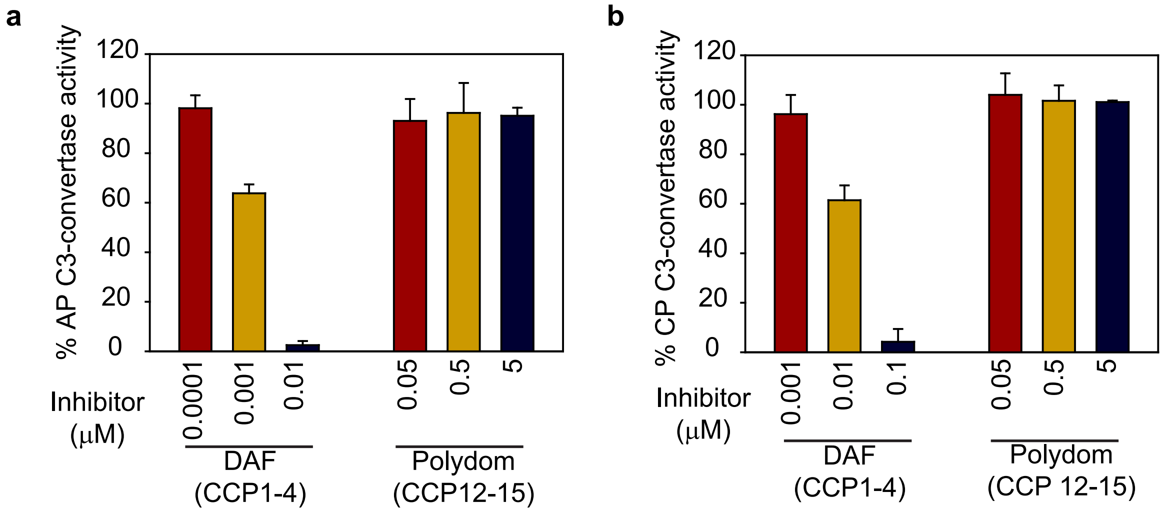

Supplementary Figure 12. Comparison of decay-acceleration activity of polydom with human regulator DAF. (a) Alternative pathway (AP) C3 convertase decay-acceleration activity. (b) Classical pathway (CP) C3 convertase decay acceleration activity. C3-convertase activity in the absence of a regulator was considered as 100% and used for normalization. Data are presented as mean ± SD of three independent experiments.

Supplementary Table 1: Primers used for construction of various RCA proteins

**Protein name**	**Primer**	**Sequence**
Polydom	Forward	5’ GGGAATTCCATATGTGTGAAAAACCTCCATCGGTTTCC 3’
	Reverse	5’ CCCAAGCTTCTTGGCAGGCATGCACTTTGGATTTG 3’
LHR-DM[4]	Forward	5’ gTggTgACCTACCgCTgCAATCCTggAAgCggAgggAgAAAgCTgTTTgAgCTTgTgggAgAACg 3’
	Reverse	5’ gATTgCAgCggTAggTCACCACTgATCCATAgTgAAAATTCTCTCTATTgTTgCTgTAgAAgTCTCC 3’
LHR-A	Forward	5’CATgCCATgggCTgCAATgCCCCAgAATggCTTC 3’
	Reverse	5’CCCAAgCTTTTTgTTAggTATAATgCACTgAggg 3’
LHR-D	Forward	5’CATgCCATgggCTgTAAAACCCCAgAgCAgTTTCC 3’
	Reverse	5’CCCAAgCTTTTTATTAgTAgAAATACACCgAggg 3’

Supplementary References

1. Kuttner-Kondo, L. *et al.* Structure-based mapping of DAF active site residues that accelerate the decay of C3 convertases. *Journal of Biological Chemistry* **282**, 18552-18562 (2007).

2. Mark, L. *et al.* The Kaposi's sarcoma-associated herpesvirus complement control protein mimics human molecular mechanisms for inhibition of the complement system. *J. Biol. Chem.* **279**, 45093-45101 (2004).

3. Pyaram, K., Kieslich, C. A., Yadav, V. N., Morikis, D. & Sahu, A. Influence of electrostatics on the complement regulatory functions of Kaposica, the complement inhibitor of Kaposi's sarcoma-associated herpesvirus. *J. Immunol.* **184**, 1956-1967 (2010).

4. Birmingham, D. J. *et al.* A CR1 polymorphism associated with constitutive erythrocyte CR1 levels affects binding to C4b but not C3b. *Immunology* **108**, 531-538 (2003).

5. Reza, M. J., Kamble, A., Ahmad, M., Krishnasastry, M. V. & Sahu, A. Dissection of functional sites in herpesvirus saimiri complement control protein homolog. *J. Virol.* **87**, 282-295 (2013).

6. Krych-Goldberg, M. *et al.* Decay accelerating activity of complement receptor type 1 (CD35). Two active sites are required for dissociating C5 convertases. *J. Biol. Chem.* **274**, 31160-31168 (1999).

7. Bresin, E. *et al.* Combined complement gene mutations in atypical hemolytic uremic syndrome influence clinical phenotype. *J. Am. Soc. Nephrol.* **24**, 475-486 (2013).

8. Liszewski, M. K. *et al.* Dissecting sites important for complement regulatory activity in membrane cofactor protein (MCP; CD46). *J. Biol. Chem.* **275**, 37692-37701 (2000).

9. Hasan, R. J. *et al.* Structure-function analysis of decay-accelerating factor: identification of residues important for binding of the Escherichia coli Dr adhesin and complement regulation. *Infect. Immun.* **70**, 4485-4493 (2002).

10. Liszewski, M. K. *et al.* Smallpox inhibitor of complement enzymes (SPICE): dissecting functional sites and abrogating activity. *J. Immunol.* **183**, 3150-3159 (2009).

11. Yadav, V. N., Pyaram, K., Mullick, J. & Sahu, A. Identification of hot spots in the variola virus complement inhibitor (SPICE) for human complement regulation. *J. Virol.* **82**, 3283-3294 (2008).

12. Gautam, A. K. *et al.* Mutational analysis of Kaposica reveals that bridging of MG2 and CUB domains of target protein is crucial for the cofactor activity of RCA proteins. *Proc. Natl. Acad. Sci. U. S. A* **112**, 12794-12799 (2015).

13. Panwar, H. S. *et al.* Molecular engineering of an efficient four-domain DAF-MCP chimera reveals the presence of functional modularity in RCA proteins. *Proc. Natl. Acad. Sci. U. S. A* **116**, 9953-9958 (2019).

14. Krych, M. *et al.* Analysis of the functional domains of complement receptor type 1 (C3b/C4b receptor, CD35) by substitution mutagenesis. *J Biol. Chem.* **269**, 13273-13278 (1994).

15. Blom, A. M., Villoutreix, B. O. & Dahlback, B. Mutations in alpha-chain of C4BP that selectively affect its factor I cofactor function. *J. Biol. Chem.* **278**, 43437-43442 (2003).

16. Williams, P. *et al.* Mapping CD55 function. The structure of two pathogen-binding domains at 1.7 A. *J. Biol. Chem.* **278**, 10691-10696 (2003).

17. Krych, M., Hauhart, R. & Atkinson, J. P. Structure-function analysis of the active sites of complement receptor type 1. *J. Biol. Chem.* **273**, 8623-8629 (1998).

18. Pechtl, I. C., Kavanagh, D., McIntosh, N., Harris, C. L. & Barlow, P. N. Disease-associated N-terminal complement factor H mutations perturb cofactor and decay-accelerating activities. *J. Biol. Chem.* **286**, 11082-11090 (2011).

19. Krych-Goldberg, M., Hauhart, R. E., Porzukowiak, T. & Atkinson, J. P. Synergy between two active sites of human complement receptor type 1 (CD35) in complement regulation: implications for the structure of the classical pathway C3 convertase and generation of more potent inhibitors. *J. Immunol.* **175**, 4528-4535 (2005).

20. Blom, A. M., Zadura, A. F., Villoutreix, B. O. & Dahlback, B. Positively charged amino acids at the interface between alpha-chain CCP1 and CCP2 of C4BP are required for regulation of the classical C3-convertase. *Mol. Immunol.* **37**, 445-453 (2000).

21. Blom, A. M., Webb, J., Villoutreix, B. O. & Dahlback, B. A cluster of positively charged amino acids in the C4BP alpha-chain is crucial for C4b binding and factor I cofactor function. *J. Biol. Chem.* **274**, 19237-19245 (1999).

22. Chaudhary, P., Hepgur, M., Sarkissian, S., Smith, R. J. & Weitz, I. C. Atypical haemolytic-uraemic syndrome due to heterozygous mutations of CFH/CFHR1-3 and complement factor H 479. *Blood Transfus.* **12**, 111-113 (2014).

23. Fremeaux-Bacchi, V. *et al.* Genetic and functional analyses of membrane cofactor protein (CD46) mutations in atypical hemolytic uremic syndrome. *J. Am. Soc. Nephrol.* **17**, 2017-2025 (2006).

24. Mohlin, F. C. *et al.* Analysis of genes coding for CD46, CD55, and C4b-binding protein in patients with idiopathic, recurrent, spontaneous pregnancy loss. *Eur. J. Immunol.* **43**, 1617-1629 (2013).

25. Kerr, H. *et al.* Disease-linked mutations in factor H reveal pivotal role of cofactor activity in self-surface-selective regulation of complement activation. *J. Biol. Chem.* **292**, 13345-13360 (2017).
