# Supplementary material for: Spatially conserved motifs in complement control protein domains determine functionality in regulators of complement activation-family proteins
Source: Commun Biol. 2019 Aug 5;2:290. doi: 10.1038/s42003-019-0529-9 (PMC6683126; doi:10.1038/s42003-019-0529-9)
Supplement: Supplementary file 2 — Description of Additional Supplementary Files [file 42003_2019_529_MOESM2_ESM.docx]

**Supplementary Data 1**: **Mammalian and viral RCA protein sequences employed for identifying novel sequence motifs.**

**Supplementary Data 2: Mutations in the motif regions of various RCA proteins^.^** Mutations marked in the red caused >3 fold decrease/increase in activity/binding from the wild type protein, while mutations marked in green showed no decrease/increase or <3 fold decrease/increase. Residues linked with diseases are marked by one asterisk and those that showed gain in activity are marked by two asterisks. Abbreviations: CR1, Complement receptor 1; C4BP, C4b binding protein; CCPH, HVS-CCPH (*Herpesvirus saimiri* – complement control protein homolog); DAF, Decay accelerating factor; FH, Factor H; KAPO, KAPOSICA (Kaposi’s sarcoma-associated herpesvirus inhibitor of complement activation); MCP, Membrane cofactor protein; SPICE, Smallpox inhibitor of complement enzymes; VCP, Vaccinia virus complement control protein. Pos refers to the position in motif.

**Supplementary Data 3: RCA protein homologs annotated as complement regulators by the 4-motif pattern search.**

**Supplementary Data 4: Raw data for figures 4c and 5e.**
