# Supplementary material for: Spatially conserved motifs in complement control protein domains determine functionality in regulators of complement activation-family proteins
Source: Commun Biol. 2019 Aug 5;2:290. doi: 10.1038/s42003-019-0529-9 (PMC6683126; doi:10.1038/s42003-019-0529-9)
Supplement: Supplementary file 8 — Reporting Summary [file 42003_2019_529_MOESM8_ESM.pdf]

natureresearch

Corresponding author(s): Arvind Sahu

Last updated by author(s): Jul 1, 2019

Reporting Summary

Nature Research wishes to improve the reproducibility of the work that we publish. This form provides structure for consistency and transparency in reporting. For further information on Nature Research policies, see Authors & Referees and the Editorial Policy Checklist.

Statistics

For all statistical analyses, confirm that the following items are present in the figure legend, table legend, main text, or Methods section.

n/a	Confirmed	
☐	☒	The exact sample size (n) for each experimental group/condition, given as a discrete number and unit of measurement
☐	☒	A statement on whether measurements were taken from distinct samples or whether the same sample was measured repeatedly
☒	☐	The statistical test(s) used AND whether they are one- or two-sided<br>*Only common tests should be described solely by name; describe more complex techniques in the Methods section.*
☒	☐	A description of all covariates tested
☒	☐	A description of any assumptions or corrections, such as tests of normality and adjustment for multiple comparisons
☐	☒	A full description of the statistical parameters including central tendency (e.g. means) or other basic estimates (e.g. regression coefficient) AND variation (e.g. standard deviation) or associated estimates of uncertainty (e.g. confidence intervals)
☒	☐	For null hypothesis testing, the test statistic (e.g. F, t, r) with confidence intervals, effect sizes, degrees of freedom and P value noted<br>*Give P values as exact values whenever suitable.*
☒	☐	For Bayesian analysis, information on the choice of priors and Markov chain Monte Carlo settings
☒	☐	For hierarchical and complex designs, identification of the appropriate level for tests and full reporting of outcomes
☒	☐	Estimates of effect sizes (e.g. Cohen's d, Pearson's r), indicating how they were calculated

Our web collection on statistics for biologists contains articles on many of the points above.

Software and code

Policy information about availability of computer code

Data collection	NCBI (www.ncbi.nlm.nih.gov/) and UniProt (www.uniprot.org/)
Data analysis	MEME and MAST (MEME suite-5.0.3, open source), MEGA-5.2 (https://www.megasoftware.net/older_versions, free for research and education), CD-hit (http://weizhong-lab.ucsd.edu/cdhit-web-server/cgi-bin/index.cgi?cmd=cd-hit), pFAM (https://pfam.xfam.org/, open source), PDB (https://www.rcsb.org/, open source), InterPro (https://www.ebi.ac.uk/interpro/, open source), pymol (vs 2.3.1, student license), Discovery Studio v 3.5 (Dassault Systèmes BIOVIA 2016, commercial), Modeller ver 9.7 available from BIOVIA (commercial), PISA (https://www.ebi.ac.uk/pdbe/pisa/, open source)

For manuscripts utilizing custom algorithms or software that are central to the research but not yet described in published literature, software must be made available to editors/reviewers. We strongly encourage code deposition in a community repository (e.g. GitHub). See the Nature Research guidelines for submitting code & software for further information.

Data

Policy information about availability of data

All manuscripts must include a data availability statement. This statement should provide the following information, where applicable:

- Accession codes, unique identifiers, or web links for publicly available datasets
- A list of figures that have associated raw data
- A description of any restrictions on data availability

The authors declare that the data supporting the findings of this study are available within the paper [and its supplementary information files]. Any other raw experimental data that support the findings of this study are available from the corresponding author upon reasonable request.

Field-specific reporting

Please select the one below that is the best fit for your research. If you are not sure, read the appropriate sections before making your selection.

☒ Life sciences ☐ Behavioural & social sciences ☐ Ecological, evolutionary & environmental sciences

For a reference copy of the document with all sections, see nature.com/documents/nr-reporting-summary-flat.pdf

Life sciences study design

All studies must disclose on these points even when the disclosure is negative.

Sample size	Criterion for choosing RCA sequences are detailed in the manuscript
Data exclusions	RCA sequences which showed more than 95% similarity were removed for unbiased motif construction
Replication	The data is individually and separately replicated three times with success
Randomization	Not applicable for our study
Blinding	Not applicable for our study

Reporting for specific materials, systems and methods

We require information from authors about some types of materials, experimental systems and methods used in many studies. Here, indicate whether each material, system or method listed is relevant to your study. If you are not sure if a list item applies to your research, read the appropriate section before selecting a response.

Materials & experimental systems

n/a	Involved in the study	
☐	☒	Antibodies
☒	☐	Eukaryotic cell lines
☒	☐	Palaeontology
☐	☒	Animals and other organisms
☐	☒	Human research participants
☒	☐	Clinical data

Methods

n/a	Involved in the study	
☒	☐	ChIP-seq
☒	☐	Flow cytometry
☒	☐	MRI-based neuroimaging

Antibodies

Antibodies used	Anti-sheep erythrocyte antibody was procured from ICN Biomedical Inc. (Irvine, CA). Cat#55806 ; lot#03176 dilution used 1:80.
Validation	The supplier has tested hemagglutination titer of the antibody. The titer was 1:3200.

Animals and other organisms

Policy information about studies involving animals; ARRIVE guidelines recommended for reporting animal research

Laboratory animals	Blood from Rabbit and Guinea Pig were obtained from Animal house facility of NCCS. The sheep blood was obtained from the local slaughterhouse.
Wild animals	Study did not involve wild animals.
Field-collected samples	Study did not involve samples collected from the field.
Ethics oversight	National Centre for Cell Sciences (NCCS).

Note that full information on the approval of the study protocol must also be provided in the manuscript.

Human research participants

Policy information about studies involving human research participants

Population characteristics	Human blood was obtained from the healthy subjects after informed consent .

Recruitment	Healthy volunteers.
Ethics oversight	National center for cell sciences (NCCS)

Note that full information on the approval of the study protocol must also be provided in the manuscript.
